# Association between osteoporosis knowledge and bone health in patients with autoimmune diseases: a cross-sectional study

**DOI:** 10.3389/fmed.2026.1741970

**Published:** 2026-02-04

**Authors:** Binbin Tang, Juxian Xian, Wenjing Zhang, Yuexiu Su, Shiao Wang, Ying Deng, Jingli Tian, Yucheng Li, Yuyue Chen, Qing Chen, Zhaoting Hu

**Affiliations:** 1 Department of Epidemiology, School of Public Health, Southern Medical University, Guangzhou, Guangdong, China; 2 Department of Health Management Center, The Third Affiliated Hospital of Southern Medical University, Guangzhou, Guangdong, China

**Keywords:** autoimmune diseases, bone mineral density, knowledge, OPAAT, osteoporosis

## Abstract

**Background:**

Osteoporosis is a common skeletal disorder among older adults, characterized by reduced bone density and increased susceptibility to fractures, particularly in individuals with autoimmune diseases. Adequate knowledge of osteoporosis and active engagement in bone-protective behaviors may help prevent its onset; however, research examining this hypothesis within autoimmune populations remains limited. This study aimed to evaluate osteoporosis knowledge levels in patients with autoimmune diseases and to investigate the relationship between osteoporosis knowledge scores and bone mineral density (BMD).

**Methods:**

This hospital-based cross-sectional study enrolled 562 participants aged 18 years and older who underwent complete dual-energy X-ray absorptiometry (DXA) scans with autoimmune diseases between March 2023 and September 2024. Latent class analysis (LCA) was applied to the four dimensions of the Chinese version of the Osteoporosis Prevention and Awareness Tool (OPAAT-C), namely symptoms, diagnostic methods, preventive measures and risk factors, to classify participants into high, moderate and low knowledge groups, and bone mass was compared across these groups.

**Results:**

This study included 562 adults with autoimmune diseases who completed the OPAAT-C (48.2% male; mean age 45.9 years). The average osteoporosis knowledge score was 12.67 ± 5.63 (57.6% of 22 points). Among 253 patients (45.0%) with low BMD, 78 (30.8%) were in the low knowledge score group based on LCA. Higher osteoporosis knowledge was significantly associated with greater lumbar spine BMD and lower osteoporosis risk. In multivariate linear regression adjusted for all covariates, higher osteoporosis knowledge scores derived from LCA were positively associated with lumbar spine BMD (*β* = 0.051; 95% CI: 0.013 to 0.088; *p* = 0.008). In multivariate logistic regression, participants in the highest knowledge quartile (Q4) had a 51.7% lower risk of low BMD compared with those in the lowest quartile (Q1) (OR = 0.481; 95% CI, 0.240 to 0.956; *p* = 0.038). Mediation analysis showed a significant indirect effect via the action score (*β* = −0.015; 95% CI: −0.031 to −0.003; *p* = 0.041), and subgroup analysis revealed a significant interaction between knowledge quartile and sex on lumbar BMD (*p* = 0.027).

**Conclusion:**

Patients with autoimmune diseases and limited osteoporosis knowledge had significantly lower bone mineral density and an increased risk of osteoporosis. Increasing physical activity and adopting healthy lifestyle behaviors can reduce this risk.

## 1 Introduction

Osteoporosis (OP) is a metabolic bone disorder characterized by reduced BMD and microstructural deterioration, increasing the risk of fragility fractures, particularly in the hip and vertebrae (1–3). Its prevalence is rising with global population aging, with a meta-analysis reporting 18.3% worldwide and a 2017 Chinese population-based study reporting 5.0% in men and 20.6% in women aged ≥40 years (4, 5).

Systemic autoimmune diseases—including rheumatoid arthritis (RA), systemic lupus erythematosus (SLE), ankylosing spondylitis (AS), and ANCA-associated vasculitis—arise from immune self-tolerance breakdown and significantly impair skeletal health (6). In RA in particular, chronic inflammation and dysregulated immune pathways contribute directly to systemic and local bone loss, with interactions between pro-inflammatory cytokines, immune cells, and bone remodeling networks driving both erosions and osteoporosis beyond classical risk factors (7). This complex osteoimmunologic interplay underscores the elevated risk of low bone mass and fracture observed in RA cohorts. Glucocorticoid-induced osteoporosis (GIOP) is a leading cause of secondary osteoporosis due to widespread glucocorticoid use in these patients (8). Osteoporosis prevalence among RA patients is 27.6% globally, 30.6% in Asian cohorts, and up to 37.7% in Chinese RA populations (9, 10). Beyond RA, AS, and SLE, a systematic review and meta-analysis reported high prevalence of osteoporosis and fractures in axial spondyloarthritis, indicating impaired bone health across inflammatory spondyloarthropathies (11).

Osteoporosis risk factors are nonmodifiable (female sex, age, genetics, ethnicity) and modifiable (physical inactivity, inadequate calcium intake, low BMI, lower education) (12).

Although effective pharmacological therapies for osteoporosis exist, many patients in routine clinical practice fail to adhere consistently to their prescribed treatment. A systematic review by Alahmari et al. (13) reported that a substantial proportion of patients discontinue therapy within the first year, with adherence rates as low as 8% in some cohorts, resulting in increased fracture risk and diminished gains in bone mineral density. Cornelissen et al. (14) further demonstrated that multicomponent interventions, particularly those combining patient education, counseling, and active patient involvement, are among the most effective strategies to enhance treatment persistence and reduce discontinuation. These findings highlight the critical role of addressing knowledge gaps in comprehensive osteoporosis management.

Interventions targeting osteoporosis knowledge can improve outcomes: a cluster randomized trial of 270 postmenopausal women improved BMD testing and treatment rates (15), and an 18-month community-based intervention in 162 older adults improved femoral neck BMD among adherent participants (16).

Evidence linking osteoporosis knowledge to bone health is mainly from middle-aged and older adults (17), postmenopausal cohorts (18), and healthcare providers (19), with autoimmune patients underrepresented. A cross-sectional study assessed osteoporosis knowledge in RA patients (20) but did not correlate knowledge with clinical BMD outcomes. Our study integrates osteoporosis knowledge assessment with DXA-derived lumbar spine and femoral neck BMD, enabling site-specific correlation analysis. A quasi-experimental study in Iranian postmenopausal women over 50 showed that combining osteoporosis knowledge with preventive behavior promotion improved lumbar spine BMD (21). Building on this, our study investigates preventive actions as mediators of the effect of osteoporosis knowledge on BMD. The aim of this study was to evaluate osteoporosis knowledge in patients with autoimmune diseases and its association with BMD.

## 2 Methods

### 2.1 Study design and participants

This cross-sectional study enrolled patients with autoimmune diseases at a general between March 2023 and September 2024. The current analysis included 562 participants aged 18 years and older with complete DXA - derived BMD data. Participants underwent in-person interviews conducted by specialist physicians.

### 2.2 Sample size calculation

Sample size calculation was performed using Cochran’s formula (22):


$$n={\left(\frac{Z_{1-\frac{\alpha }{2}}}{\delta }\right)}^{2}\times p\times q$$

n denotes the initially estimated sample size. $Z_{1-\frac{\alpha }{2}}$ denotes the Z value corresponding to the chosen confidence level, where *α* represents the type I error rate and is set at 0.05; a 95% confidence level corresponds to Z = 1.96. *δ* represents the allowable margin of error and is set at 0.05. *p* denotes the anticipated prevalence; when unknown, p is set to 0.5, yielding the maximum variance and thus the most conservative sample size estimate. q is calculated as 1 − p . With p set to 0.5, the most conservative estimated sample size was 384. After accounting for an anticipated 30% missing data rate, the minimum required sample size was increased to 550 questionnaires.

### 2.3 Chinese version of Osteoporosis Prevention and Awareness Tool

The Osteoporosis Prevention and Awareness Tool (OPAAT) is a psychometrically robust instrument originally developed by Lai et al. (23) and later refined by Toh et al. (24) for Asian populations. In our study, the OPAAT was cross-culturally translated into Chinese, with eight items removed and the retained content reorganized into four dimensions: symptoms (A1-A5), diagnostic methods (B1-B5), prevention measures (C1-C5), and risk factors (D1-D7), enhancing its cultural applicability for Chinese populations. The final Chinese version (OPAAT-C) consists of 22 items assessed using dichotomous scoring (1 = correct, 0 = incorrect/uncertain), with total scores (range 0–22) positively correlating with osteoporosis knowledge levels.

Two analytical strategies were applied to evaluate osteoporosis knowledge. First, quartile-based categorization was applied to the total osteoporosis knowledge score. Second, LCA was performed based on four knowledge domains: symptoms, diagnostic methods, preventive measures, and risk factors. Preventive measures had two levels, while the other factors had three levels. The LCA uses multiple observed categorical variables to generate an unmeasured variable (i.e., latent variable) with a set of mutually exclusive latent classes (25). In our study, three latent classes were identified, representing high, moderate, and low osteoporosis knowledge score groups based on item-response probabilities. Supplementary Tables 3, 4 described the data collection and LCA of participants.

### 2.4 Bone mineral density assessment

BMD (measured in grams/cm^2^) was assessed using DXA. The mean BMD for the anteroposterior length of L1 to L4 was calculated and used for lumbar spine BMD reporting. The left hip (or right hip if left hip arthroplasty or metal object injection was performed) was routinely scanned to report femoral neck BMD. T-scores were calculated as (measured BMD - young adult mean BMD) / standard deviation. According to the World Health Organization guidelines, osteoporosis (T-score ≤ − 2.5), osteopenia (−2.5 < T-score < −1), and normal bone mass (T-score ≥ − 1) (26). A classification system based on BMD levels was implemented, in which participants with normal parameters at both sites were classified into the normal BMD group, while those with osteopenia or osteoporosis at either site were classified into the low BMD group (27).

### 2.5 Assessment of covariates

Covariates were classified into four domains based on existing evidence (28–32): (1) demographic characteristics (age, sex, BMI, education, occupation), (2) dietary/lifestyle factors (cigarette/alcohol use, tea/milk/regular calcium intake, physical activity levels), and (3) clinical profiles (types of autoimmune disease, autoimmune disease duration, use of glucocorticoids, history of fractures). Data were collected using structured questionnaires. Age was dichotomized according to WHO criteria (33): <60 vs. ≥60 years (older adults). Self-reported variables included gender, weight, and height. Education was classified as less than junior high school or junior high school or above, and occupation as farmers/non-farmers. Smoking status and alcohol consumption were dichotomized into current users (“Yes”) versus former or never users (“No”). Tea intake and milk consumption were defined as ≥1 cup/week and <1 cup/week (34, 35). Regular calcium intake was defined as ≥3 times per week through supplements. Sunshine duration was classified into two categories based on total daily sunlight exposure: ≤60 min per day and > 60 min per day. Physical activity (PA) was assessed using the International Physical Activity Questionnaire short form (IPAQ-SF), and participants were asked to report the frequency and usual duration of each type of activity performed for at least 10 min at a time. Exercise intensity was quantified in terms of metabolic equivalents (METs). Standard MET values were used: vigorous (8 METs), moderate (4 METs), and walking (3.3 METs). Weekly PA energy expenditure (MET-min/week) was calculated as: MET value × duration (minutes per day) × frequency (days per week). Total weekly MET-minutes were derived from the sum of all activity intensities. PA levels were classified as low (<600 MET-min/week and <5 d/w of PA at three intensities), moderate (600–2,999 and ≥5), or high (≥3,000 and ≥7) (36). Types of autoimmune diseases were categorized as RA, AS, SLE, and “Other.” The “Other” category comprised several less common autoimmune diseases. Because the number of patients with each of these conditions was small, these diagnoses were combined into a single category for statistical analysis. The detailed list of all autoimmune diseases and the corresponding number of cases are shown in Supplementary Table 1. The duration of autoimmune disease, use of glucocorticoids, and history of fractures were self-reported by the patients.

### 2.6 Statistical analysis

#### 2.6.1 Baseline analysis

Categorical variables were reported as number (percentage%), and continuous variables as mean ± standard deviation (SD). In our primary analysis, covariate distributions were compared using the *t*-test for continuous variables and the Pearson *χ^2^* test for categorical variables.

#### 2.6.2 Linear and logistic regression analysis

Linear and logistic regression models were used to analyze the associations between osteoporosis knowledge score and BMD. Multivariable linear regression models were constructed with lumbar spine (L1 to L4) and femoral neck BMD values as dependent variables and osteoporosis knowledge score groups (quartile and LCA) as predictors, adjusting for covariates including age, gender, BMI, education level, occupation, smoking status, alcohol/milk consumption, tea/calcium intake, sunlight exposure, physical activity, types of autoimmune disease, autoimmune disease duration, use of glucocorticoid, and history of fractures. Logistic regression was performed to calculate the odds ratio (OR) for low BMD among BMD groups, with the aforementioned adjustments.

#### 2.6.3 Mediation analysis

The osteoporosis action score was constructed as a weighted composite of seven self-reported bone-protective actions: smoking status, alcohol consumption, tea intake, milk consumption, regular calcium intake, sunshine duration, and physical activity levels (they are all the action-related factors among the covariates previously included). Each behavior was coded as present or absent and weighted by its standardized coefficient (*β*) from a multivariable logistic regression predicting the risk of low BMD. The final score was computed by summing the weighted behavior components, with higher values indicating greater adherence to recommended protective behaviors. Details are provided in Supplementary Table 2.

Osteoporosis knowledge was assessed with a validated questionnaire encompassing four domains: symptoms, diagnostic methods, preventive measures, and risk factors. LCA was applied to participants’ responses to identify two distinct knowledge levels—low and high. To adjust for potential confounding in class assignment, education levels, occupation, and types of autoimmune disease were included as covariates in the LCA model.

To assess whether the relationship between osteoporosis knowledge score and BMD levels was mediated by osteoporosis action score, we conducted a mediation analysis using Model 4 of the PROCESS macro (version 2024.6) in R. In this model, osteoporosis knowledge score group (low vs. high) was entered as the independent variable (X), the weighted osteoporosis action score as the mediator (M), and the risk of low BMD/lumbar spine BMD/femur neck BMD as the binary outcome (Y). Covariates included age groups, sex, BMI groups, autoimmune disease duration, use of glucocorticoid, and history of fracture. Lastly, mediation analysis was performed with the fourth model of PROCESS for SPSS macro. A confidence interval of 95% and 10,000 bootstrap resamples was executed.

#### 2.6.4 Subgroup analyses

Subgroup analyses were conducted based on age, gender, education, occupation, smoking status, alcohol consumption, tea intake, milk consumption and regular calcium intake. The study cohort achieved a 100% response rate on the OPAAT, and all covariates had ≤5% missing data. Missing data were imputed using the random forest imputation method implemented in the mice package of R software. We used a restricted cubic spline analysis to capture the dose-effect relationship connection with osteoporosis knowledge score and the risk of low BMD/lumbar spine BMD/femur neck BMD.

#### 2.6.5 Sensitivity analyses

No independent external validation cohort was available for this study. To assess the robustness of our findings internally, we performed sensitivity analyses including multiple imputation (MI), complete-case analysis (CCA), and single mean/mode imputation (SI). Complete-case analysis (CCA), excluding any subject with a missing covariate; and (2) single mean/mode imputation (SI), imputing continuous variables by their mean and categorical variables by their mode. We compared the direction and significance of effects across all three missing-data strategies in our regression models.

All analyses were conducted using R Studio (R v4.5.0). Two-sided *p* values < 0.05 were considered significant.

## 3 Results

### 3.1 Participant characteristics

Table 1 presents the baseline characteristics of participants with autoimmune diseases. Among 562 participants (mean age 45.84 years; 63.5% female), 309 (55.0%) were in the normal BMD group, while 253 (45.0%) were in the low BMD group. Participants in the low BMD group were more likely to be older, female, have a BMI < 18.5 kg/m^2^, lower education levels, work in farming occupations, have RA, longer disease durations, and a history of fractures. Quartile-based categorization of osteoporosis knowledge scores divided participants into four groups: Q1 (*n* = 144, 25.6%), Q2 (*n* = 145, 25.8%), Q3 (*n* = 168, 29.9%), and Q4 (*n* = 105, 18.7%). LCA stratified participants into three knowledge groups: high (*n* = 252, 44.8%), moderate (*n* = 175, 31.1%), and low (*n* = 135, 24.0%). χ^2^ analysis showed significant differences in BMD status distribution across knowledge groups (*p* < 0.001). Detailed information on the specific diagnoses included in the “Other autoimmune diseases” category is presented in Supplementary Table 1. Due to the small number of cases for each individual disease, these conditions were analyzed as a combined group.

**Table 1:** Baseline characteristics of participants.

Variables	Overall (*N* = 562)	BMD levels		*P* -value
		Normal BMD	Low BMD	
Total	562 (100)	309 (55.0)	253 (45.0)	
Age, years	45.84 ± 13.95	41.15 ± 12.48	51.56 ± 13.52	**<0.001**
Age groups				**<0.001**
<60 years	446 (79.4)	279 (90.3)	167 (66.0)	
≥60 years	116 (20.6)	30 (9.7)	86 (34.0)	
Sex				0.078
Male	205 (36.5)	123 (39.8)	82 (32.4)	
Female	357 (63.5)	186 (60.2)	171 (67.6)	
BMI, kg/m2	22.88 ± 3.91	23.71 ± 3.85	21.87 ± 3.74	**<0.001**
BMI groups				**<0.001**
<18.5	65 (11.6)	17 (5.5)	48 (19.0)	
18.5–23.9	298 (53.0)	153 (49.5)	145 (57.3)	
24–27.9	143 (25.4)	99 (32.0)	44 (17.4)	
≥28	56 (10.0)	40 (12.9)	16 (6.3)	
Education				**<0.001**
Less than junior high school	303 (53.9)	194 (62.8)	109 (43.1)	
Junior high school or above	259 (46.1)	115 (37.2)	144 (56.9)	
Occupation				**<0.001**
Farmers	161 (28.6)	68 (22.0)	93 (36.8)	
Non farmers	401 (71.4)	241 (78.0)	160 (63.2)	
Smoking status				0.823
Yes	104 (18.5)	56 (18.1)	48 (19.0)	
No	458 (81.5)	253 (81.9)	205 (81.0)	
Alcohol consumption				0.473
Yes	85 (15.1)	50 (16.2)	35 (13.8)	
No	477 (84.9)	259 (83.8)	218 (86.2)	
Tea intake, /week				0.178
> = 1 cup	229 (40.7)	134 (43.4)	95 (37.5)	
<1 cup	333 (59.3)	175 (56.6)	158 (62.5)	
Milk consumption, /week				0.447
> = 1 cup	268 (47.7)	152 (49.2)	116 (45.8)	
<1 cup	294 (52.3)	157 (50.8)	137 (54.2)	
Regular calcium intake				0.200
Yes	214 (38.1)	110 (35.6)	104 (41.1)	
No	348 (61.9)	199 (64.4)	149 (58.9)	
Sunshine duration, /day				0.124
≤60 min	403 (71.7)	230 (74.4)	173 (68.4)	
>60 min	159 (28.3)	79 (25.6)	80 (31.6)	
Physical activity levels				0.611
Low intensity	96 (17.1)	57 (18.4)	39 (15.4)	
Moderate intensity	208 (37.0)	113 (36.6)	95 (37.5)	
High intensity	258 (45.9)	139 (45.0)	119 (47.0)	
Types of autoimmune disease				**<0.001**
Rheumatoid arthritis	158 (28.1)	66 (21.4)	92 (36.4)	
Ankylosing spondylitis	136 (24.2)	87 (28.2)	49 (19.4)	
Systemic lupus erythematosus	61 (10.9)	45 (14.6)	16 (6.3)	
Other	207 (36.8)	111 (35.9)	96 (37.9)	
Disease duration, years	5.21 ± 5.21	4.70 ± 4.75	5.83 ± 5.66	**0.012**
Use of glucocorticoid				0.822
Yes	113 (20.1)	61 (19.7)	52 (20.6)	
No	449 (79.9)	248 (80.3)	201 (79.4)	
History of fractures				**<0.001**
Yes	77 (13.7)	26 (8.4)	51 (20.2)	
No	485 (86.3)	283 (91.6)	202 (79.8)	
Osteoporosis knowledge score	12.67 ± 5.63	13.57 ± 5.29	11.57 ± 5.85	**<0.001**
Osteoporosis knowledge score groups (quartile)				**<0.001**
Q1 (≤9)	144 (25.6)	63 (20.4)	81 (32.0)	
Q2 (9–14)	145 (25.8)	73 (23.6)	72 (28.5)	
Q3 (14–17)	168 (29.9)	99 (32.0)	69 (27.3)	
Q4 (17–21)	105 (18.7)	74 (23.9)	31 (12.3)	
Osteoporosis knowledge score groups (LCA)				**<0.001**
Low	135 (24.0)	57 (18.4)	78 (30.8)	
Moderate	175 (31.1)	93 (30.1)	82 (32.4)	
High	252 (44.8)	159 (51.5)	93 (36.8)	

BMI, body mass index; DXA, dual-energy X-ray absorptiometry; LCA, latent class analysis.

*p*-values < 0.05 are presented in bold.

Categorical variables are presented as counts (frequencies), and continuous variables are reported as means ± standard deviation (SD).

In the types of autoimmune diseases, “Other” comprises all diseases included in the study except rheumatoid arthritis, ankylosing spondylitis, and systemic lupus erythematosus; see Supplementary Table 1 for the full list of specific disease names. Osteoporosis knowledge score groups (LCA): Participants were categorized into low, moderate, and high groups based on LCA.

### 3.2 Osteoporosis knowledge scores

Table 2 presents the total and subscale scores of the OPAAT-C. The overall OPAAT-C score was 12.67 ± 5.63 (mean ± SD) out of a possible 22 points. Participants showed the highest proficiency in preventive measures, with the highest subscale score (4.07 ± 1.48) and mean item score (0.82 ± 0.30). In contrast, knowledge of risk factors was the weakest, with the lowest subscale score (2.66 ± 2.00) and mean item score (0.38 ± 0.29). Supplementary Table 2 provides the correct and incorrect response rates for all 22 OPAAT-C items. The statement “To prevent falls, comfortable shoes with a good grip should be worn” had the highest correct response rate (85.8%), while “A bone mineral density test is high in radiation” had the highest incorrect response rate (75.3%).

**Table 2:** Scoring profile of the Chinese version of Osteoporosis Prevention and Awareness Tool: total score and domain-specific performance.

OPAAT-C	Range	Item (mean ± SD)	Item average (mean ± SD)
Symptoms	0–5	2.90 ± 1.66	0.58 ± 0.33
Diagnostic method	0–5	3.04 ± 1.75	0.61 ± 0.35
Preventive measure	0–5	4.07 ± 1.48	0.82 ± 0.30
Risk factors	0–7	2.66 ± 2.00	0.38 ± 0.29
Total score	0–22	12.67 ± 5.63	0.58 ± 0.26

OPAAT-C, Chinese Version of Osteoporosis Prevention and Awareness Tool.

The tool comprises 22 items, categorized into four dimensions: A1-A5 (symptoms), B1-B5 (diagnostic methods), C1-C5 (preventive measures), and D1-D7 (risk factors).

### 3.3 Association between groups with different osteoporosis knowledge scores and BMD at specific sites

The associations between osteoporosis knowledge score groups and BMD at different bone sites were separately analyzed among participants (Table 3). LCA revealed a positive association between lumbar spine BMD and the group with high osteoporosis knowledge scores (*β* = 0.051, 95% CI: 0.013 to 0.088, *p* = 0.008, *P* for trend = 0.010); however, no association was observed for femoral neck BMD. In the quartile analysis, no multivariate linear relationship was observed between osteoporosis knowledge score groups (quartiles) and lumbar spine BMD or femoral neck BMD.

**Table 3:** Multiple linear regression results for dependent variables lumbar spine BMD and femur neck BMD.

Variables	BMD (gm/cm^2^)			
	Lumbar spine		Femur neck	
	*β* (95% CI)	*P*-Value	*β* (95% CI)	*P*-Value
Osteoporosis knowledge score	0.003 (0.000, 0.006)	**0.043**	0.002 (0.000, 0.004)	**0.035**
Osteoporosis knowledge score groups				
Quartile 1	1[reference]		1[reference]	
Quartile 2	0.026 (−0.013, 0.065)	0.188	0.015 (−0.015, 0.046)	0.315
Quartile 3	0.025 (−0.014, 0.065)	0.208	0.019 (−0.012, 0.050)	0.221
Quartile 4	0.040 (−0.007, 0.086)	0.096	0.034 (−0.002, 0.071)	0.064
*P* for trend	0.118		0.072	
Osteoporosis knowledge score groups (LCA)				
Low	1[reference]		1[reference]	
Moderate	0.034 (−0.003, 0.071)	0.074	0.028 (−0.001, 0.056)	0.061
High	0.051 (0.013, 0.088)	**0.008**	0.027 (−0.002, 0.056)	0.068
*P* for trend	**0.010**		0.098	

LCA, latent class analysis, BMD, bone mass density (gm/cm^2^); *β*, coefficient; CI, confidence interval.

Linear regression adjusted for age, sex, and BMI, education, occupation, smoking status, alcohol consumption, tea intake, milk consumption, regular calcium intake, sunshine duration, physical activity levels, autoimmune disease duration, types of autoimmune disease, use of glucocorticoid, history of fractures.

Data are presented as *β* (95% CI), and *p*-value. *p*-values < 0.05 were marked in bold.

### 3.4 Association between osteoporosis knowledge score groups and bone mass level

Osteoporosis knowledge groups (quartile) showed significant negative associations with BMD levels across all models (Models 1–3; *p* < 0.05), suggesting that higher knowledge levels were linked to a reduced risk of low BMD (Table 4). In the quartile-based classification, participants with the highest osteoporosis knowledge scores (Q4) had 51.7% lower odds of low BMD compared to those in the lowest score group (Q1) (Model 3: OR = 0.481, 95% CI: 0.240 to 0.956, *p* = 0.038, *P* for trend = 0.051), this association was not observed In the LCA -based classification.

**Table 4:** Associations of osteoporosis knowledge score and the risk of low BMD.

Variables	Model 1		Model 2		Model 3	
	OR (95% CI)	*P*-value	OR (95% CI)	*P*-value	OR (95% CI)	*P*-value
Osteoporosis knowledge score	0.938 (0.910, 0.967)	**<0.001**	0.967 (0.932, 1.002)	0.067	0.970 (0.931, 1.010)	0.140
Osteoporosis knowledge score groups						
Quartile 1	1[reference]		1[reference]		1[reference]	
Quartile 2	0.767 (0.482, 1.218)	0.262	0.873 (0.516, 1.475)	0.612	0.886 (0.506, 1.551)	0.672
Quartile 3	0.542 (0.344, 0.849)	**0.008**	0.753 (0.444, 1.275)	0.291	0.796 (0.451, 1.405)	0.430
Quartile 4	0.326 (0.189, 0.551)	**<0.001**	0.490 (0.263, 0.903)	**0.023**	0.481 (0.240, 0.956)	**0.038**
*P* for trend	**<0.001**		**0.025**		0.051	
Osteoporosis knowledge score groups (LCA)						
Low	1[reference]		1[reference]		1[reference]	
Moderate	0.644 (0.409, 1.011)	0.057	0.780 (0.466, 1.302)	0.343	0.793 (0.464, 1.352)	0.394
High	0.427 (0.278, 0.653)	**<0.001**	0.671 (0.403, 1.116)	0.124	0.674 (0.390, 1.161)	0.155
*P* for trend	**<0.001**		0.128		0.158	

LCA, latent class analysis, CI, confidence interval, OR, odd ratio.

Osteoporosis knowledge score groups were generated through latent class analysis using information on four OPAAT-C dimensions: osteoporosis symptoms, diagnostic method, preventive measures and risk factors.

Data are presented as OR, 95% CI, and *p*-value. *p*-values < 0.05 were marked in bold.

Model 1 was not controlled for any covariates.

Model 2 was adjusted for age, sex, and BMI.

Model 3 was adjusted for age, sex, BMI, education, occupation, smoking status, alcohol consumption, tea intake, milk consumption, regular calcium intake, sunshine duration, physical activity levels, autoimmune disease duration, types of autoimmune disease, use of glucocorticoid, history of fractures.

### 3.5 Findings from restricted cubic spline analyses

The osteoporosis knowledge weight score was constructed as a weighted composite of four dimensions of OPAAT-C: symptoms, diagnostic methods, preventive measures and risk factors. Each dimension was coded as present or absent and weighted by its standardized coefficient (*β*) from a multivariable logistic regression predicting the risk of low BMD. Details are provided in Supplementary Table 7.

**Figure 1:**
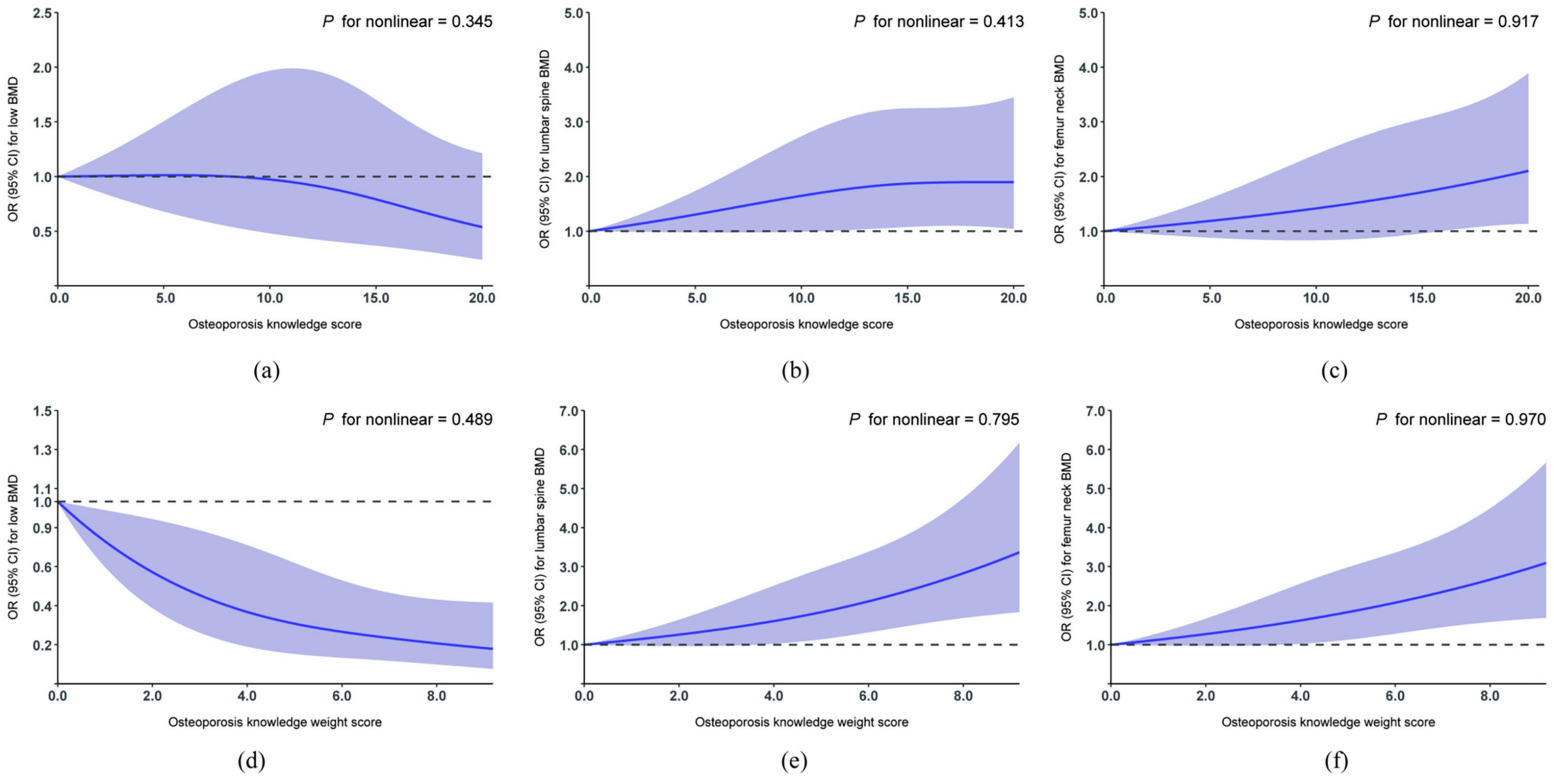
Restricted cubic spline regression analysis of osteoporosis knowledge score with the risk of low BMD/lumbar spine BMD/femur neck BMD. BMD, bone mineral density. Multivariable-adjusted ORs for the presence of the risk of low BMD **(a)**, lumbar spine BMD **(b)**, and femur neck BMD **(c)** based on restricted cubic spines with 3 knots of osteoporosis knowledge score. All models adjusted for age, sex, BMI, education, occupation, smoking status, alcohol consumption, tea intake, milk consumption, regular calcium intake, sunshine duration, physical activity levels, autoimmune disease duration, types of autoimmune disease, use of glucocorticoid, history of fractures. Multivariable-adjusted ORs for the presence of the risk of low BMD **(d)**, lumbar spine BMD **(e)**, and femur neck BMD **(f)** based on restricted cubic spines with 3 knots of osteoporosis knowledge weight score. Standardized weights were calculated as the proportion of each logistic regression coefficient (*β*) relative to the total sum of all βs in the multivariable model predicting the risk of low BMD. All models adjusted for age, sex, BMI, education, occupation, smoking status, alcohol consumption, tea intake, milk consumption, regular calcium intake, sunshine duration, physical activity levels, autoimmune disease duration, types of autoimmune disease, use of glucocorticoid, history of fractures.

Multivariable-adjusted restricted cubic spline analysis revealed no significant non-linear associations between the osteoporosis knowledge score and the risk of low BMD, lumbar spine BMD, or femur neck BMD; similar findings were observed for the osteoporosis knowledge weight score (Figure 1).

### 3.6 Subgroup analysis and sensitivity analyses

In the subgroup analysis, there was a significant interaction between osteoporosis knowledge score quartiles and lumbar spine (L1-L4) BMD by sex (*p* = 0.027) (Supplementary Tables 5, 6). Supplementary Tables 8, 9 present the ORs, *β* coefficients, and *p*values from multivariable logistic regression models of osteoporosis knowledge score and low BMD, and from multiple linear regression models of osteoporosis knowledge score with lumbar spine and femoral neck BMD, using multiple imputation (MI), complete-case analysis (CCA), and single mean/mode imputation (SI). Supplementary Tables 10, 11 extend these comparisons to the four OPAAT-C dimensions. These analyses show minor variations in score estimates across methods but consistent direction and significance, indicating robustness to missing-data assumptions.

### 3.7 Mediation analysis

**Figure 2:**
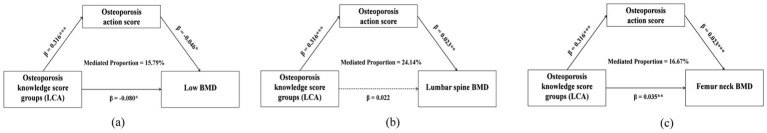
Model showing the influence of osteoporosis knowledge score on the risk of low BMD/lumbar spine BMD/femur neck BMD and the mediating effect of osteoporosis action score. BMD, bone mineral density; LCA, latent class analysis. Mediation models were specified with the osteoporosis knowledge score as the independent variable, the osteoporosis action score as the mediator, and three outcomes: **(a)** Risk of low BMD, **(b)** Lumbar spine BMD, and **(c)** Femoral neck BMD. All models adjusted for age groups, sex, and BMI groups, autoimmune disease duration, use of glucocorticoid, and history of fractures. ***p < 0.001, **p < 0.01, *p < 0.05.

Model 4 of the PROCESS macro was applied with 10,000 bootstrap samples and a 95% confidence interval to test whether the osteoporosis action score mediated the relationship between osteoporosis knowledge and the risk of low BMD, as indicated by a significant indirect effect (*β* = −0.015, 95% CI: −0.031 to −0.003, *p* = 0.041). The direct effect of osteoporosis knowledge on BMD status remained significant after accounting for the mediator (*β* = −0.080, 95% CI: −0.156 to −0.002, *p* = 0.041), and the total effect on low BMD risk was also significant (*β* = −0.095, 95% CI = −0.170 to −0.016, *p* = 0.016). An indirect effect was observed in the relationship between osteoporosis knowledge and lumbar spine BMD (*β* = 0.007, 95% CI: 0.002 to 0.015, *p* = 0.021), while the direct and total effects were not statistically significant. A significant indirect effect was also found in the relationship between osteoporosis knowledge and femur neck BMD (*β* = 0.007, 95% CI: 0.003 to 0.013, *p* = 0.006). The direct effect on femur neck BMD remained significant after adjusting for the mediator (β = 0.035, 95% CI = 0.013 to 0.056, *p* = 0.002), and the total effect was also significant (*β* = 0.042, 95% CI: 0.019 to 0.064, *p* < 0.001). Refer to Figure 2.

## 4 Discussion

In this cross-sectional analysis of 562 patients with autoimmune diseases, we employed the OPAAT-C questionnaire, a translated and revised version of the original OPAAT, to evaluate osteoporosis knowledge (Cronbach’s *α* = 0.898; CFI = 0.930; TLI = 0.920). The questionnaire covers four domains: symptoms, diagnostic methods, preventive measures, and risk factors, with scores for the risk factors domain significantly lower than others. We assessed the relationship between osteoporosis knowledge and BMD at the lumbar spine and femoral neck. In multivariable linear regression models adjusted for all covariates, LCA showed lumbar spine BMD was positively associated with the high knowledge group. Multivariable logistic regression using quartiles indicated that participants with high knowledge had a lower risk of low BMD. Subgroup analysis revealed a significant interaction between knowledge quartiles and sex on lumbar spine BMD. Mediation analysis showed osteoporosis knowledge directly and indirectly reduced the risk of low BMD by promoting protective osteoporosis behaviors.

Participants had a moderate level of osteoporosis knowledge (57.6% of 22 points), consistent with studies in China (37) and Malaysia (17). Analysis of OPAAT-C responses showed population-wide differences, emphasizing the need for targeted educational interventions. Understanding of osteoporosis risk factors was lower than knowledge of preventive measures, consistent with prior Chinese studies (37). Participants scored highest on preventive items such as “To prevent falls, shoes with good traction should be used” (84.5%), “Poor vision can lead to falls” (80.0%), and “Calcium supplements may help prevent osteoporosis” (75.9%).

Several misconceptions need addressing. First, 75.3% mistakenly believed BMD testing involves significant radiation, although most modalities (DXA, HR-pQCT, pQCT, QUS) involve minimal exposure except QCT (38, 39). Second, 61.7% believed calcium supplementation contributes to kidney stones, despite evidence in RA patients showing calcium and vitamin D3 prevent steroid-induced spinal bone loss (40), and meta-analyses confirming fracture prevention with good adherence (41). Third, 68.9% believed exercise harms bones, whereas studies in early RA (42) and SLE (43) patients show physical activity increases BMD and improves HRQoL. Structured education programs are needed to dispel misconceptions and promote preventive practices.

Higher osteoporosis knowledge scores were positively associated with lumbar spine BMD in multivariable linear regression by LCA and emerged as protective against low BMD in logistic regression by quartiles. Previous studies reported variable results: the Silesia Osteo Active cohort (44) found a weak link with femoral neck BMD but not lumbar spine, while studies in Polish (45) and Iranian (46) populations suggest knowledge improves bone health through better lifestyle practices. Subgroup analyses showed sex differences: women had higher knowledge scores and rated their knowledge more satisfactory than men (2).

Educational interventions can enhance BMD. Trials in premenopausal Tasmanian women (47), elderly patients (48), postmenopausal women in India (49), and high-risk older adults (50) showed that combining osteoporosis education with diet, exercise, or rehabilitation improved lumbar spine and femoral neck BMD. In juvenile RA, a knowledge- and behavior-based intervention increased calcium intake and improved bone mass (51). Our mediation analysis supports that knowledge indirectly benefits BMD by promoting protective behaviors. However, in adult autoimmune patients, RCTs focusing solely on osteoporosis knowledge education with BMD outcomes are lacking, as most studies target pharmacologic or exercise interventions (52–54).

Future studies should use randomized controlled designs to assess systematic knowledge interventions on BMD and fracture risk in adults with autoimmune diseases.

This study has several limitations. First, the cross-sectional design precludes establishing causal relationships or providing longitudinal data for patients with autoimmune diseases. Second, single-center sampling may not represent heterogeneous autoimmune disease manifestations. Third, although we adjusted for sociodemographic, lifestyle/dietary, and clinical covariates, residual confounding (e.g., psychological stress, environmental exposures) may persist. Fourth, self-reported data including anthropometric measures (height/weight) introduce potential recall and reporting biases. Fifth, questionnaire complexity might cause misclassification of osteoporosis knowledge levels. Sixth, several less common autoimmune diseases were represented by small sample sizes and were grouped into an “Other” category, limiting disease-specific analyses beyond RA, AS, and SLE. Seventh, external validation was not performed due to the lack of an independent cohort. Validation in multicenter or population-based cohorts would strengthen the generalizability of our findings. We conducted multiple sensitivity analyses, including multiple imputation and complete-case analysis, which showed largely consistent trends. Future studies should replicate these results in independent cohorts to confirm their robustness. Eighth, we did not specifically assess patients’ awareness of the potential adverse effects of glucocorticoids, commonly used in these diseases, on bone because the Chinese version of the OPAAT questionnaire used in this study has a fixed set of questions and does not include this topic. Future studies could include such questions to better capture medication-related bone health knowledge.

## 5 Conclusion

This cross-sectional study indicates that patients with autoimmune diseases have a relatively low understanding of osteoporosis risk factors, underscoring the need for targeted public education to address this deficiency. Furthermore, osteoporosis knowledge levels correlated with BMD. Higher osteoporosis knowledge scores were positively associated with BMD at the femoral neck, and as scores increased, the risk of low BMD among autoimmune patients declined. In addition, osteoporosis action score acts as a mediator between osteoporosis knowledge and the risk of BMD.

## Data Availability

The raw data supporting the conclusions of this article will be made available by the authors, without undue reservation.

## Glossary

BMD: Bone mineral density
DXA: Dual-energy X-ray absorptiometry
OPAAT-C: the Chinese version of the Osteoporosis Prevention and Awareness Tool
OP: Osteoporosis
RA: Rheumatoid arthritis
SLE: Systemic lupus erythematosus
AS: Ankylosing spondylitis
GIOP: Glucocorticoid-induced osteoporosis
BMI: Body mass index
LCA: Latent class analysis
IPAQ-SF: Physical Activity Questionnaire short form
METs: Metabolic equivalents
CCA: Complete-case analysis
HR-pQCT: High-resolution peripheral quantitative computed tomography
pQCT: Peripheral quantitative
QCT: Quantitative CT
QUS: Quantitative ultrasound
HRQoL: Health-related quality of life
SD: Standard deviation
CI: Confidence interval
