# Supplementary material for: Association between osteoporosis knowledge and bone health in patients with autoimmune diseases: a cross-sectional study
Source: Front Med (Lausanne). 2026 Feb 4;13:1741970. doi: 10.3389/fmed.2026.1741970 (PMC12913584; doi:10.3389/fmed.2026.1741970)
Supplement: Supplementary file 1 [file Table_1.DOCX]

**Supplementary Material**

Supplementary Table 1 lists all other diseases—apart from rheumatoid arthritis, ankylosing spondylitis, and systemic lupus erythematosus—identified from the medical records of our study cohort.

Symptoms, diagnostic methods, preventive measureand risk factors represent distinct aspects of the Chinese Version of the Osteoporosis Prevention and Awareness Tool (OPAAT-C). These four parameters were used to calculate an overall knowledge score for the participants. Supplementary Table 2 presents the accuracy and error rates for each of the 22 items in the OPAAT-C. Each dimension was categorized into low, moderate, and high groups based on tertiles, while preventive measures were categorized using the median.

Latent class analysis was performed with varying numbers of latent classes to identify the optimal model. Model selection was based on the Akaike Information Criterion (AIC), Bayesian Information Criterion (BIC), entropy, the Lo-Mendell-Rubin likelihood ratio test (LMR), and class prevalence. Since the six-class model failed to converge, only models with ≤5 classes were retained. Fit indices for the 1-5 class models are presented in Supplementary Table 2. The three-class model demonstrated the best fit, with the lowest AIC/BIC values, acceptable entropy (0.849), a statistically significant LMR result (P < 0.05), and well-balanced class probabilities (44.0%, 34.4%, 21.5%).

Supplementary Table 3 presents the mean posterior probabilities, prevalence, latent class proportions, and item-response probabilities for the 3-class models. The mean posterior probability, which reflects the uncertainty of posterior classification, was also considered during model selection, with values ≥0.7 indicating acceptable uncertainty. Item-response probabilities, derived from posterior probabilities, were used to define latent classes. We also evaluated the mean posterior probabilities to aid in model selection. All mean posterior probabilities for the three- and four- class solution were >0.77, mean posterior probabilities of latent classes 3 from the five-latent-class solution were 0.68 which were less than 0.70 (Supplementary Table 4). Therefore, mean posterior probabilities alone were not sufficient for selecting the optimal classification.

All mean posterior probabilities exceeded 0.82 in the three-latent-class model. After a thorough evaluation of model selection statistics (AIC/BIC, entropy, LMR), posterior classification certainty, the interpretation of latent classes, and parsimony, the three-latent-class solution was determined to be optimal in terms of classification uncertainty. Participants were therefore stratified into high, moderate, and low osteoporosis knowledge groups. One type of osteoporosis knowledge group was derived through latent class analysis (LCA) of the four OPAAT-C dimension score.

Supplementary Tables 5 and 6 present the results of the subgroup analyses.

Supplementary Table 7 presents the coding criteria, multivariable logistic regression coefficients (β), and standardized weights for four dimensions of OPAAT-C—symptoms, diagnostic methods, preventive measures and risk factors—used to derive the osteoporosis knowledge weight score.

Supplementary Tables 8 and 9 present sensitivity analyses of multivariable logistic models for osteoporosis knowledge score and low BMD alongside multiple linear models for osteoporosis knowledge score with lumbar spine and femoral neck BMD, using multiple imputation (MI), complete-case analysis (CCA), and single mean/mode imputation (SI). Supplementary Tables 10 and 11 extend these comparisons to the four osteoporosis knowledge score dimensions, applying the same three missing-data strategies.

Supplementary Table 12 presents the coding criteria, multivariable logistic regression coefficients (*β*), and standardized weights for seven behaviors—smoking status; alcohol consumption; tea and milk intake; regular calcium supplementation; sunshine duration; and physical activity—used to derive the osteoporosis action score.

Supplementary Table 1. Detailed diseases of the “types of autoimmune disease”.

| Category | Specific names of autoimmune diseases (number) | Total count |
| --- | --- | --- |
| Total |  | 562 |
| Main autoimmune diseases | Rheumatoid arthritis (158), Ankylosing spondylitis (136), Systemic lupus erythematosus (61) | 355 |
| Arthritis | Gouty arthritis (34), Generalized osteoarthritis (21), Undifferentiated arthritis (19), Osteoarthritis unspecified (16), Spondyloarthropathy (10), Sacroiliitis (3), Gonarthrosis (4), Polyarthritis (5), Spinal arthropathy (2), Bilateral knee osteoarthritis (3), Psoriatic arthritis (2), Reactive arthritis (1), Psoriatic arthropathy (1), Synovitis (1), Bilateral knee synovitis (1), Left coxarthrosis (1) | 124 |
| Connective tissue disease | Undifferentiated CTD (41), Sjögren's syndrome (6), Dermatomyositis (6), Diffuse systemic sclerosis (6), Systemic sclerosis (5), Polymyositis (4), Localized scleroderma (1), Adult-onset Still disease (1), Plaque psoriasis (1), Severe plaque psoriasis (1), Erythema nodosum (1), Allergic contact dermatitis (1) | 74 |
| Rare/single-case autoimmune diseases | SAPHO syndrome (2), Acute rheumatic fever (1), Rheumatoid nodules (1), Diffuse idiopathic skeletal hyperostosis (1), Osteitis condensans ilii (1), Autoimmune hemolytic anemia (1) | 7 |
| Systemic Vasculitides | ANCA-associated vasculitis (1), Systemic vasculitis (1) | 2 |

Supplementary Table 2. Frequency and percentages of answer accuracy and error rate of the Chinese Version of Osteoporosis Prevention and Awareness Tool.

| OPAAT-C items | Correct answer (%) | Wrong answer (%) |
| --- | --- | --- |
| A1: Osteoporosis occurs because bone is removed faster than it is formed. | 176 (31.3%) | 386 (68.7%) |
| A2: Osteoporosis can result in back pain. | 358 (63.7%) | 204 (36.3%) |
| A3: Loss of height or hunchback. | 327 (58.2%) | 235 (41.8%) |
| A4: Loss of mobility (unable to move around myself). | 372 (66.2%) | 190 (33.8%) |
| A5: The recommended daily intake for calcium in women above 50 years of age is 1000mg. | 396 (70.5%) | 166 (29.5%) |
| B1: Makes bones weaker, more brittle and more likely to break (fracture). | 401 (71.4%) | 161 (28.6%) |
| B2: Osteoporosis and osteoarthritis are different names we can use to describe the same disease. | 254 (45.2%) | 308 (54.8%) |
| B3: A bone mineral density test is used to diagnose osteoporosis. | 347 (61.7%) | 215 (38.3%) |
| B4: I do not need a bone mineral density test unless I fracture my bones. | 353 (62.8%) | 209 (37.2%) |
| B5: A bone mineral density test should be performed monthly to monitor bone loss. | 354 (63%) | 208 (37%) |
| C1: Calcium supplements can help prevent osteoporosis. | 436 (77.6%) | 126 (22.4%) |
| C2: Foods such as milk, tofu, anchovies (ikan bilis), yellow dhal and spinach are rich in calcium. | 451 (80.2%) | 111 (19.8%) |
| C3: You can obtain your recommended daily intake of vitamin D via exposing your skin to sunlight for about 15 minutes a day. | 466 (82.9%) | 96 (17.1%) |
| C4: To prevent falls, comfortable shoes with a good grip should be used. | 482 (85.8%) | 80 (14.2%) |
| C5: Poor vision may lead to falls. | 456 (81.1%) | 106 (18.9%) |
| D1: A bone mineral density test is high in radiation. | 139 (24.7%) | 423 (75.3%) |
| D2: It is too late to increase calcium intake after the age of 50. | 155 (27.6%) | 407 (72.4%) |
| D3: The regular dose of calcium supplements can cause kidney stones. | 215 (38.3%) | 347 (61.7%) |
| D4: Increasing coffee and tea intake can help in osteoporosis prevention. | 278 (49.5%) | 284 (50.5%) |
| D5: Exercise will wear out bones. | 175 (31.1%) | 387 (68.9%) |
| D6: Certain medications (such as sleeping tablets or high blood pressure medications) may reduce the risk of falling. | 231 (41.1%) | 331 (58.9%) |
| D7: Being underweight helps prevent osteoporosis. | 300 (53.4%) | 262 (46.6%) |

OPAAT-C: Chinese Version of Osteoporosis Prevention and Awareness Tool

The tool comprises 22 items, categorized into four dimensions: A1-A5 (symptoms), B1-B5 (diagnostic methods), C1-C5 (preventive measures), and D1-D7 (risk factors).

Supplementary Table 3. Fit statistics for Models with One -Five latent Classes.

| Number of latent classes | AIC | BIC | Entropy | LMR(P) | Prevalence |
| --- | --- | --- | --- | --- | --- |
| 1 | 4340.687 | 4371.008 |  |  | 1 |
| 2 | 3872.993 | 3937.965 | 0.862 | ＜0.001 | 0.636/0.364 |
| 3 | 3847.066 | 3946.691 | 0.849 | ＜0.001 | 0.440/0.344/0.215 |
| 4 | 3848.050 | 3982.327 | 0.845 | 0.073 | 0.392/0.251/0.210/0.146 |
| 5 | 3852.383 | 4021.312 | 0.843 | 0.202 | 0.269/0.227/0.219/0.144/0.141 |

AIC = Akaike information criterion, BIC = Bayesian information criterion, LMR = Lo-Mendell-Rubin.

In latent class analysis (LCA), prevalence refers to the estimated proportion of the population assigned to each latent class, while entropy quantifies the certainty of these assignments by evaluating the distribution of posterior probabilities across individuals.

Supplementary Table 4. Mean posterior probabilities, classification probability of latent classes, and item-response probabilities in models with three latent classes in the OPAAT-C score.

| Item | Latent class 1 | Latent class 2 | Latent class 3 | Latent class 4 | Latent class 5 |
| --- | --- | --- | --- | --- | --- |
| Three-latent-class solution | | | | | |
| MeanPP | 0.85 | 0.85 | 0.82 | NA | NA |
| Prevalence | 0.44 | 0.34 | 0.22 | NA | NA |
| Symptoms (low) | 0.09 | **0.42** | **0.85** | NA | NA |
| Symptoms1 (moderate) | **0.68** | 0.36 | 0.15 | NA | NA |
| Symptoms1 (high) | 0.23 | 0.22 | 0.00 | NA | NA |
| Diagnostic method (low) | 0.00 | 0.39 | **1.00** | NA | NA |
| Diagnostic method (moderate) | 0.46 | **0.55** | 0.00 | NA | NA |
| Diagnostic method (high) | **0.54** | 0.06 | 0.00 | NA | NA |
| Preventive measures (low) | 0.18 | 0.39 | **0.87** | NA | NA |
| Preventive measures (high) | **0.82** | **0.61** | 0.13 | NA | NA |
| Risk factors (low) | 0.04 | 0.37 | **0.93** | NA | NA |
| Risk factors (moderate) | **0.56** | **0.51** | 0.07 | NA | NA |
| Risk factors (high) | 0.40 | 0.21 | 0.00 | NA | NA |
| four-latent-class solution | | | | | |
| MeanPP | 0.82 | 0.83 | 0.91 | 0.90 | NA |
| Prevalence | 0.39 | 0.25 | 0.21 | 0.15 | NA |
| Symptoms (low) | 0.08 | **0.50** | **0.84** | 0.22 | NA |
| Symptoms1 (moderate) | **0.66** | 0.27 | 0.16 | **0.65** | NA |
| Symptoms1 (high) | 0.26 | 0.23 | 0.00 | 0.13 | NA |
| Diagnostic method (low) | 0.00 | **0.50** | **0.99** | 0.10 | NA |
| Diagnostic method (moderate) | 0.46 | 0.49 | 0.01 | **0.59** | NA |
| Diagnostic method (high) | **0.54** | 0.01 | 0.00 | 0.31 | NA |
| Preventive measures (low) | 0.00 | 0.24 | **0.93** | **1.00** | NA |
| Preventive measures (high) | **1.00** | **0.76** | 0.07 | 0.00 | NA |
| Risk factors (low) | 0.05 | **0.45** | **0.92** | 0.12 | NA |
| Risk factors (moderate) | **0.56** | 0.44 | 0.08 | **0.63** | NA |
| Risk factors (high) | 0.39 | 0.11 | 0.00 | 0.25 | NA |
| five-latent-class solution | | | | | |
| MeanPP | 0.78 | 0.89 | 0.63 | 0.94 | 0.73 |
| Prevalence | 0.38 | 0.22 | 0.17 | 0.14 | 0.09 |
| Symptoms (low) | 0.13 | **0.84** | 0.35 | **0.72** | 0.00 |
| Symptoms1 (moderate) | **0.55** | 0.16 | **0.65** | 0.16 | **0.67** |
| Symptoms1 (high) | 0.32 | 0.00 | 0.00 | 0.12 | 0.33 |
| Diagnostic method (low) | 0.22 | **0.96** | 0.00 | **0.63** | 0.00 |
| Diagnostic method (moderate) | **0.70** | 0.04 | 0.40 | 0.35 | 0.43 |
| Diagnostic method (high) | 0.08 | 0.00 | **0.60** | 0.02 | **0.57** |
| Preventive measures (low) | **0.51** | **1.00** | 0.45 | 0.00 | 0.00 |
| Preventive measures (high) | 0.49 | 0.00 | **0.55** | **1.00** | **1.00** |
| Risk factors (low) | 0.26 | **0.88** | 0.01 | **0.56** | 0.04 |
| Risk factors (moderate) | **0.62** | 0.11 | **0.63** | 0.34 | **0.52** |
| Risk factors (high) | 0.12 | 0.01 | 0.36 | 0.10 | 0.44 |

MeanPP=mean posterior probability; NA=not available.

The maximum item-response probabilities for each latent class are highlighted in bold.

The mean posterior probability (MPP) was used to reflect the uncertainty in posterior classification. Item-response probabilities, defined as posterior probabilities, were used to characterize the latent classes. Classification probability represented the proportion of the population in each latent class. Osteoporosis symptoms 1–3, diagnostic methods 1–3, and risk factors 1–3 were categorized into low, moderate, and high groups based on tertiles, while preventive measures were categorized based on the median.

Supplementary Table 5. Subgroup Analysis of osteoporosis knowledge score groups (quartile) and lumbar spine and femoral neck BMD.

| Subgroups | Osteoporosis knowledge score | BMD (g/cm^2^) | | | |
| --- | --- | --- | --- | --- | --- |
|  |  | Lumbar spine (L1-L4) | | Femur neck | |
|  |  | *β* (95% CI) | *P* for interaction | *β* (95% CI) | *P* for interaction |
| Age groups |  |  | 0.424 |  | 0.197 |
| <60 years | Osteoporosis knowledge score (std.) | 0.019 (-0.018, 0.055) |  | 0.014 (-0.014, 0.041) |  |
|  | Osteoporosis knowledge score groups |  |  |  |  |
|  | Quartile 1 | 1[reference] |  | 1[reference] |  |
|  | Quartile 2 | 0.043 (-0.049, 0.136) |  | 0.008 (-0.063, 0.078) |  |
|  | Quartile 3 | 0.035 (-0.060, 0.131) |  | -0.003 (-0.075, 0.069) |  |
|  | Quartile 4 | 0.011 (-0.097, 0.119) |  | 0.032 (-0.050, 0.113) |  |
| ≥60 years | Osteoporosis knowledge score (std.) | 0.014 (-0.002, 0.031) |  | **0.015 (0.002, 0.028)** |  |
|  | Osteoporosis knowledge score groups |  |  |  |  |
|  | Quartile 1 | 1[reference] |  | 1[reference] |  |
|  | Quartile 2 | 0.027 (-0.013, 0.067) |  | 0.024 (-0.008, 0.057) |  |
|  | Quartile 3 | 0.029 (-0.016, 0.074) |  | 0.028 (-0.008, 0.065) |  |
|  | Quartile 4 | 0.035 (-0.012, 0.081) |  | 0.033 (-0.004, 0.071) |  |
| Sex |  |  | **0.027** |  | 0.513 |
| Male | Osteoporosis knowledge score (std.) | 0.015 (-0.014, 0.044) |  | **0.025 (0.003, 0.047)** |  |
|  | Osteoporosis knowledge score groups |  |  |  |  |
|  | Quartile 1 | 1[reference] |  | 1[reference] |  |
|  | Quartile 2 | 0.035 (-0.039, 0.109) |  | 0.026 (-0.031, 0.082) |  |
|  | Quartile 3 | 0.035 (-0.037, 0.108) |  | **0.057 (0.002, 0.112)** |  |
|  | Quartile 4 | 0.044 (-0.043, 0.130) |  | **0.071 (0.005, 0.137)** |  |
| Female | Osteoporosis knowledge Z-score | 0.014 (-0.004, 0.032) |  | 0.010 (-0.005, 0.025) |  |
|  | Osteoporosis knowledge score groups |  |  |  |  |
|  | Quartile 1 | 1[reference] |  | 1[reference] |  |
|  | Quartile 2 | 0.030 (-0.012, 0.073) |  | 0.022 (-0.012, 0.057) |  |
|  | Quartile 3 | 0.015 (-0.034, 0.063) |  | 0.021 (-0.018, 0.061) |  |
|  | Quartile 4 | 0.036 (-0.017, 0.089) |  | 0.036 (-0.008, 0.079) |  |
| Education |  |  | 0.935 |  | 0.811 |
| Less than junior high school | Osteoporosis knowledge score (std.) | 0.016 (-0.005, 0.036) |  | 0.009 (-0.007, 0.026) |  |
|  | Osteoporosis knowledge score groups |  |  |  |  |
|  | Quartile 1 | 1[reference] |  | 1[reference] |  |
|  | Quartile 2 | **0.058 (0.006, 0.109)** |  | -0.004 (-0.046, 0.038) |  |
|  | Quartile 3 | **0.054 (0.001, 0.107)** |  | 0.003 (-0.040, 0.047) |  |
|  | Quartile 4 | 0.042 (-0.018, 0.102) |  | 0.029 (-0.020, 0.079) |  |
| Junior high school or above | Osteoporosis knowledge score (std.) | 0.012 (-0.007, 0.031) |  | **0.015 (0.000, 0.030)** |  |
|  | Osteoporosis knowledge score groups |  |  |  |  |
|  | Quartile 1 | 1[reference] |  | 1[reference] |  |
|  | Quartile 2 | 0.020 (-0.031, 0.070) |  | 0.011 (-0.028, 0.051) |  |
|  | Quartile 3 | **0.057 (0.006, 0.108)** |  | 0.024 (-0.016, 0.063) |  |
|  | Quartile 4 | 0.020 (-0.040, 0.080) |  | 0.018 (-0.029, 0.065) |  |
| Occupation |  |  | 0.266 |  | 0.325 |
| Non farmers | Osteoporosis knowledge score (std.) | 0.013 (-0.004, 0.031) |  | **0.016 (0.001, 0.030)** |  |
|  | Osteoporosis knowledge score groups |  |  |  |  |
|  | Quartile 1 | 1[reference] |  | 1[reference] |  |
|  | Quartile 2 | 0.018 (-0.026, 0.062) |  | 0.029 (-0.007, 0.064) |  |
|  | Quartile 3 | 0.016 (-0.034, 0.065) |  | 0.016 (-0.025, 0.056) |  |
|  | Quartile 4 | 0.034 (-0.016, 0.083) |  | **0.043 (0.003, 0.083)** |  |
| Farmers | Osteoporosis knowledge score (std.) | 0.016 (-0.012, 0.044) |  | 0.012 (-0.008, 0.032) |  |
|  | Osteoporosis knowledge score groups |  |  |  |  |
|  | Quartile 1 | 1[reference] |  | 1[reference] |  |
|  | Quartile 2 | 0.042 (-0.028, 0.112) |  | -0.022 (-0.072, 0.029) |  |
|  | Quartile 3 | 0.022 (-0.052, 0.096) |  | -0.025 (-0.079, 0.028) |  |
|  | Quartile 4 | 0.032 (-0.045, 0.110) |  | 0.037 (-0.019, 0.093) |  |
| Smoking status |  |  | 0.144 |  | 0.286 |
| Yes | Osteoporosis knowledge score (std.) | -0.004 (-0.048, 0.040) |  | **0.028 (0.001, 0.055)** |  |
|  | Osteoporosis knowledge score groups |  |  |  |  |
|  | Quartile 1 | 1[reference] |  | 1[reference] |  |
|  | Quartile 2 | -0.050 (-0.162, 0.062) |  | 0.000 (-0.069, 0.069) |  |
|  | Quartile 3 | -0.017 (-0.139, 0.106) |  | 0.032 (-0.044, 0.108) |  |
|  | Quartile 4 | -0.020 (-0.140, 0.099) |  | 0.067 (-0.007, 0.141) |  |
| No | Osteoporosis knowledge score (std.) | 0.016 (-0.001, 0.033) |  | 0.011 (-0.003, 0.025) |  |
|  | Osteoporosis knowledge score groups |  |  |  |  |
|  | Quartile 1 | 1[reference] |  | 1[reference] |  |
|  | Quartile 2 | 0.019 (-0.021, 0.059) |  | 0.019 (-0.014, 0.051) |  |
|  | Quartile 3 | 0.016 (-0.030, 0.062) |  | 0.016 (-0.022, 0.054) |  |
|  | Quartile 4 | 0.031 (-0.017, 0.079) |  | 0.032 (-0.007, 0.071) |  |
| Alcohol consumption |  |  | 0.999 |  | 0.887 |
| Yes | Osteoporosis knowledge score (std.) | 0.007 (-0.035, 0.050) |  | 0.017 (-0.017, 0.052) |  |
|  | Osteoporosis knowledge score groups |  |  |  |  |
|  | Quartile 1 | 1[reference] |  | 1[reference] |  |
|  | Quartile 2 | -0.029 (-0.148, 0.090) |  | 0.059 (-0.037, 0.155) |  |
|  | Quartile 3 | 0.006 (-0.103, 0.116) |  | 0.030 (-0.058, 0.118) |  |
|  | Quartile 4 | -0.025 (-0.156, 0.105) |  | 0.024 (-0.081, 0.129) |  |
| No | Osteoporosis knowledge score (std.) | 0.013 (-0.004, 0.032) |  | 0.013 (-0.001, 0.026) |  |
|  | Osteoporosis knowledge score groups |  |  |  |  |
|  | Quartile 1 | 1[reference] |  | 1[reference] |  |
|  | Quartile 2 | 0.031 (-0.011, 0.073) |  | 0.013 (-0.020, 0.046) |  |
|  | Quartile 3 | 0.022 (-0.021, 0.065) |  | 0.019 (-0.015, 0.052) |  |
|  | Quartile 4 | 0.037 (-0.013, 0.088) |  | 0.037 (-0.003, 0.076) |  |
| Tea intake, /week |  |  | 0.329 |  | 0.566 |
| >=1 cup | Osteoporosis knowledge score (std.) | 0.006 (-0.021, 0.033) |  | 0.002 (-0.019, 0.022) |  |
|  | Osteoporosis knowledge score groups |  |  |  |  |
|  | Quartile 1 | 1[reference] |  | 1[reference] |  |
|  | Quartile 2 | 0.043 (-0.025, 0.110) |  | 0.042 (-0.009, 0.094) |  |
|  | Quartile 3 | 0.025 (-0.047, 0.097) |  | 0.023 (-0.032, 0.077) |  |
|  | Quartile 4 | 0.005 (-0.077, 0.087) |  | 0.015 (-0.047, 0.077) |  |
| <1 cup | Osteoporosis knowledge score (std.) | **0.022 (0.003, 0.042)** |  | **0.021 (0.005, 0.037)** |  |
|  | Osteoporosis knowledge score groups |  |  |  |  |
|  | Quartile 1 | 1[reference] |  | 1[reference] |  |
|  | Quartile 2 | 0.012 (-0.037, 0.060) |  | 0.002 (-0.036, 0.040) |  |
|  | Quartile 3 | 0.027 (-0.022, 0.076) |  | 0.024 (-0.014, 0.063) |  |
|  | Quartile 4 | **0.060 (0.001, 0.119)** |  | **0.050 (0.004, 0.096)** |  |
| Milk consumption, /week |  |  | 0.540 |  | 0.899 |
| >=1 cup | Osteoporosis knowledge score (std.) | 0.011 (-0.012, 0.033) |  | 0.006 (-0.011, 0.023) |  |
|  | Osteoporosis knowledge score groups |  |  |  |  |
|  | Quartile 1 | 1[reference] |  | 1[reference] |  |
|  | Quartile 2 | -0.006 (-0.061, 0.048) |  | 0.005 (-0.036, 0.047) |  |
|  | Quartile 3 | -0.017 (-0.080, 0.046) |  | -0.006 (-0.054, 0.043) |  |
|  | Quartile 4 | 0.006 (-0.058, 0.071) |  | 0.017 (-0.032, 0.066) |  |
| <1 cup | Osteoporosis knowledge score (std.) | 0.019 (-0.003, 0.041) |  | **0.021 (0.003, 0.038)** |  |
|  | Osteoporosis knowledge score groups |  |  |  |  |
|  | Quartile 1 | 1[reference] |  | 1[reference] |  |
|  | Quartile 2 | 0.016 (-0.038, 0.071) |  | -0.006 (-0.049, 0.036) |  |
|  | Quartile 3 | 0.040 (-0.015, 0.094) |  | 0.026 (-0.017, 0.069) |  |
|  | Quartile 4 | 0.042 (-0.028, 0.113) |  | 0.043 (-0.012, 0.098) |  |
| Regular calcium intake |  |  | 0.816 |  | 0.697 |
| Yes | Osteoporosis knowledge score (std.) | 0.005 (-0.019, 0.030) |  | 0.015 (-0.004, 0.033) |  |
|  | Osteoporosis knowledge score groups |  |  |  |  |
|  | Quartile 1 | 1[reference] |  | 1[reference] |  |
|  | Quartile 2 | 0.017 (-0.046, 0.079) |  | 0.027 (-0.019, 0.073) |  |
|  | Quartile 3 | 0.013 (-0.050, 0.075) |  | 0.045 (0.000, 0.091) |  |
|  | Quartile 4 | 0.012 (-0.061, 0.085) |  | 0.052 (-0.001, 0.105) |  |
| No | Osteoporosis knowledge score (std.) | 0.017 (-0.004, 0.038) |  | 0.012 (-0.004, 0.029) |  |
|  | Osteoporosis knowledge score groups |  |  |  |  |
|  | Quartile 1 | 1[reference] |  | 1[reference] |  |
|  | Quartile 2 | 0.023 (-0.028, 0.074) |  | 0.020 (-0.021, 0.060) |  |
|  | Quartile 3 | 0.024 (-0.030, 0.078) |  | 0.010 (-0.033, 0.053) |  |
|  | Quartile 4 | 0.048 (-0.017, 0.112) |  | 0.033 (-0.018, 0.084) |  |

Abbreviation: BMD, bone mass density (gm/cm^2^); β, coefficient; CI, confidence interval.

Osteoporosis Knowledge Score (std.) = (raw score - mean)/SD, yielding Z-scores (mean = 0, SD = 1).

Linear regression adjusted for age, sex, BMI, education, occupation, smoking status, alcohol consumption, tea intake, milk consumption, regular calcium intake, sunshine duration, physical activity levels, autoimmune disease duration, types of autoimmune disease, use of glucocorticoid, history of fractures.

Data are presented as β (95% CI) , and *P*-value. *P*-values < 0.05 were marked in bold.

Supplementary Table 6. Subgroup Analysis of osteoporosis knowledge score groups (quartile) and the risk of low BMD.

| Subgroups | Osteoporosis knowledge score | Model 3 | Subgroups | Model 3 | *P* for interaction |
| --- | --- | --- | --- | --- | --- |
|  |  | OR (95% CI) |  | OR (95% CI) |  |
| Age groups |  |  |  |  | 0.302 |
| <60 years | Osteoporosis knowledge score (std.) | 0.716 (0.369, 1.326) | ≥60 years | 0.836 (0.657, 1.063) |  |
|  | Osteoporosis knowledge score groups |  |  |  |  |
|  | Quartile 1 | 1[reference] |  | 1[reference] |  |
|  | Quartile 2 | 1.243 (0.279, 5.484) |  | 0.914 (0.510, 1.639) |  |
|  | Quartile 3 | 1.334 (0.268, 6.624) |  | 0.702 (0.362, 1.355) |  |
|  | Quartile 4 | 0.671 (0.118, 3.584) |  | 0.510 (0.252, 1.018) |  |
| Sex |  |  |  |  | 0.365 |
| Male | Osteoporosis knowledge score (std.) | 0.677 (0.444, 1.015) | Female | 0.916 (0.680, 1.232) |  |
|  | Osteoporosis knowledge score groups |  |  |  |  |
|  | Quartile 1 | 1[reference] |  | 1[reference] |  |
|  | Quartile 2 | 0.540 (0.183, 1.553) |  | 1.175 (0.597, 2.327) |  |
|  | Quartile 3 | 0.531 (0.184, 1.492) |  | 0.821 (0.379, 1.784) |  |
|  | Quartile 4 | **0.191 (0.049, 0.686)** |  | 0.697 (0.295, 1.639) |  |
| Education |  |  |  |  | 0.189 |
| Less than junior high school | Osteoporosis knowledge score (std.) | 0.956 (0.674, 1.352) | Junior high school or above | **0.753 (0.569, 0.991)** |  |
|  | Osteoporosis knowledge score groups |  |  |  |  |
|  | Quartile 1 | 1[reference] |  | 1[reference] |  |
|  | Quartile 2 | 1.163 (0.483, 2.809) |  | 0.873 (0.430, 1.772) |  |
|  | Quartile 3 | 1.111 (0.452, 2.731) |  | 0.551 (0.263, 1.141) |  |
|  | Quartile 4 | 0.925 (0.350, 2.460) |  | 0.461 (0.188, 1.089) |  |
| Occupation |  |  |  |  | 0.884 |
| Non farmers | Osteoporosis knowledge score (std.) | 0.799 (0.612, 1.040) | Farmers | 0.887 (0.564, 1.384) |  |
|  | Osteoporosis knowledge score groups |  |  |  |  |
|  | Quartile 1 | 1[reference] |  | 1[reference] |  |
|  | Quartile 2 | 0.977 (0.520, 1.843) |  | 1.883 (0.570, 6.642) |  |
|  | Quartile 3 | 0.918 (0.443, 1.903) |  | 2.416 (0.686, 9.112) |  |
|  | Quartile 4 | **0.462 (0.217, 0.972)** |  | 0.631 (0.186, 2.140) |  |
| Smoking status |  |  |  |  | 0.279 |
| Yes | Osteoporosis knowledge score (std.) | 0.545 (0.278, 1.005) | No | 0.896 (0.694, 1.155) |  |
|  | Osteoporosis knowledge score groups |  |  |  |  |
|  | Quartile 1 | 1[reference] |  | 1[reference] |  |
|  | Quartile 2 | 2.552 (0.553, 12.666) |  | 1.140 (0.628, 2.081) |  |
|  | Quartile 3 | 0.711 (0.116, 4.135) |  | 1.008 (0.511, 1.999) |  |
|  | Quartile 4 | 0.229 (0.035, 1.291) |  | 0.644 (0.308, 1.342) |  |
| Alcohol consumption |  |  |  |  | 0.862 |
| Yes | Osteoporosis knowledge score (std.) | 0.724 (0.335, 1.503) | No | 0.857 (0.663, 1.106) |  |
|  | Osteoporosis knowledge score groups |  |  |  |  |
|  | Quartile 1 | 1[reference] |  | 1[reference] |  |
|  | Quartile 2 | 1.363 (0.158, 11.697) |  | 0.839 (0.453, 1.553) |  |
|  | Quartile 3 | 1.226 (0.167, 9.602) |  | 0.810 (0.435, 1.509) |  |
|  | Quartile 4 | 0.243 (0.018, 2.485) |  | 0.538 (0.250, 1.143) |  |
| Tea intake, /week |  |  |  |  | 0.905 |
| >=1 cup | Osteoporosis knowledge score (std.) | 0.870 (0.585, 1.291) | <1 cup | 0.795 (0.587, 1.074) |  |
|  | Osteoporosis knowledge score groups |  |  |  |  |
|  | Quartile 1 | 1[reference] |  | 1[reference] |  |
|  | Quartile 2 | 0.739 (0.286, 1.887) |  | 0.969 (0.455, 2.069) |  |
|  | Quartile 3 | 0.712 (0.256, 1.956) |  | 0.714 (0.340, 1.494) |  |
|  | Quartile 4 | 0.461 (0.133, 1.534) |  | 0.460 (0.185, 1.122) |  |
| Milk consumption, /week |  |  |  |  | 0.733 |
| >=1 cup | Osteoporosis knowledge score (std.) | 0.906 (0.641, 1.283) | <1 cup | 0.766 (0.556, 1.051) |  |
|  | Osteoporosis knowledge score groups |  |  |  |  |
|  | Quartile 1 | 1[reference] |  | 1[reference] |  |
|  | Quartile 2 | 1.393 (0.629, 3.128) |  | 0.977 (0.453, 2.107) |  |
|  | Quartile 3 | 0.914 (0.355, 2.369) |  | 0.907 (0.417, 1.975) |  |
|  | Quartile 4 | 0.786 (0.298, 2.070) |  | 0.381 (0.132, 1.055) |  |
| Regular calcium intake |  |  |  |  | 0.642 |
| Yes | Osteoporosis knowledge score (std.) | **0.667 (0.449, 0.973)** | No | 0.940 (0.696, 1.274) |  |
|  | Osteoporosis knowledge score groups |  |  |  |  |
|  | Quartile 1 | 1[reference] |  | 1[reference] |  |
|  | Quartile 2 | 0.682 (0.263, 1.742) |  | 1.074 (0.517, 2.248) |  |
|  | Quartile 3 | **0.346 (0.131, 0.880)** |  | 1.297 (0.599, 2.837) |  |
|  | Quartile 4 | 0.374 (0.121, 1.112) |  | 0.542 (0.207, 1.396) |  |

Abbreviation: CI, confidence interval, OR, odd ratio.

Osteoporosis knowledge score groups was generated through latent class analysis using information on four OPAAT-C dimensions: osteoporosis symptoms, diagnostic method, preventive measures and risk factors.

Osteoporosis Knowledge Score (std.) = (raw score - mean)/SD, yielding Z-scores (mean = 0, SD = 1).

Data are presented as OR, 95% CI, and *P*-value. *P*-values < 0.05 were marked in bold.
Multivariate logistic regression (Model 3) was adjusted for age, sex, BMI, education, occupation, smoking status, alcohol consumption, tea intake, milk consumption, regular calcium intake, sunshine duration, physical activity levels, autoimmune disease duration, types of autoimmune disease, use of glucocorticoid, history of fractures.

Supplementary Table 7. Construction of the osteoporosis knowledge weight score.

| Items | Coding criteria | Logistic regression *β* | Standardized weight |
| --- | --- | --- | --- |
| Symptoms | correct = 1, incorrect/uncertain = 0 | -0.012 | 0.065 |
| Diagnostic method | correct = 1, incorrect/uncertain = 0 | 0.262 | -1.416 |
| Preventive measure | correct = 1, incorrect/uncertain = 0 | -0.304 | 1.634 |
| Risk factors | correct = 1, incorrect/uncertain = 0 | -0.313 | 0.708 |

Standardized weights were calculated as the proportion of each logistic regression coefficient (β) relative to the total sum of all βs in the multivariable model predicting the risk of low BMD.

Multivariate logistic regression was adjusted for age, sex, BMI, education, occupation, smoking status, alcohol consumption, tea intake, milk consumption, regular calcium intake, sunshine duration, physical activity levels, autoimmune disease duration, types of autoimmune disease, use of glucocorticoid, history of fractures.

Supplementary Table 8. Sensitivity analysis of multivariable logistic regression of osteoporosis knowledge score and the risk of low BMD across multiple imputation (MI), complete-case analysis (CCA), and single mean/mode imputation (SI).

| Variables | Model 3 (MI) | |  | Model 3 (CCA) | |  | Model 3 (SI) | |
| --- | --- | --- | --- | --- | --- | --- | --- | --- |
|  | OR (95% CI) | *P*-value |  | OR (95% CI) | *P*-value |  | OR (95% CI) | *P*-value |
| Osteoporosis knowledge score | 0.970 (0.931, 1.010) | 0.140 |  | 0.970 (0.926, 1.017) | 0.205 |  | 0.968 (0.929, 1.007) | 0.109 |
| Osteoporosis knowledge score groups |  |  |  |  |  |  |  |  |
| Quartile 1 | 1[reference] | |  | 1[reference] | |  | 1[reference] | |
| Quartile 2 | 0.886 (0.506, 1.551) | 0.672 |  | 1.127 (0.596, 2.138) | 0.713 |  | 0.883 (0.504, 1.545) | 0.663 |
| Quartile 3 | 0.796 (0.451, 1.405) | 0.430 |  | 0.857 (0.461, 1.598) | 0.627 |  | 0.779 (0.444, 1.368) | 0.385 |
| Quartile 4 | 0.481 (0.240, 0.956) | **0.038** |  | 0.554 (0.256, 1.184) | 0.129 |  | 0.466 (0.233, 0.923) | **0.030** |
| *P* for trend | 0.051 | |  | 0.120 | |  | **0.039** | |
| Osteoporosis knowledge score groups (LCA) |  |  |  |  |  |  |  |  |
| Low | 1[reference] | |  | 1[reference] | |  | 1[reference] | |
| Moderate | 0.793 (0.464, 1.352) | 0.394 |  | 0.693 (0.384, 1.242) | 0.219 |  | 0.852 (0.500, 1.449) | 0.553 |
| High | 0.674 (0.390, 1.161) | 0.155 |  | 0.781 (0.411, 1.481) | 0.447 |  | 0.708 (0.409, 1.225) | 0.216 |
| *P* for trend | 0.158 | |  | 0.533 | |  | 0.209 | |
| Osteoporosis knowledge weight score | 0.831 (0.757, 0.909) | **<0.001** |  | 0.824 (0.728, 0.928) | **0.002** |  | 0.823 (0.748, 0.903) | **<0.001** |
| Osteoporosis knowledge weight score groups |  |  |  |  |  |  |  |  |
| Quartile 1 | 1[reference] | |  | 1[reference] | |  | 1[reference] | |
| Quartile 2 | 0.756 (0.427, 1.332) | 0.333 |  | 0.767 (0.403, 1.454) | 0.418 |  | 0.783 (0.442, 1.384) | 0.400 |
| Quartile 3 | 0.514 (0.288, 0.909) | **0.023** |  | 0.595 (0.310, 1.139) | 0.118 |  | 0.548 (0.307, 0.969) | **0.039** |
| Quartile 4 | 0.369 (0.204, 0.658) | **<0.001** |  | 0.428 (0.219, 0.825) | **0.012** |  | 0.371 (0.206, 0.661) | **<0.001** |
| *P* for trend | **<0.001** | |  | **0.008** | |  | **<0.001** | |

Abbreviation: MI, multiple imputation, CCA, complete-case analysis, SI, single mean/mode imputation, LCA, latent class analysis, CI, confidence interval, OR, odd ratio.

Osteoporosis knowledge score groups was generated through latent class analysis using information on four OPAAT-C dimensions: osteoporosis symptoms, diagnostic method, preventive measures and risk factors. Osteoporosis knowledge weight score were calculated as the proportion of each logistic regression coefficient (β) relative to the total sum of all βs in the multivariable model predicting low BMD.

Data are presented as OR, 95% CI, and *P*-value. *P*-values < 0.05 were marked in bold.

Model 3 was adjusted for age, sex, BMI, education, occupation, smoking status, alcohol consumption, tea intake, milk consumption, regular calcium intake, sunshine duration, physical activity levels, autoimmune disease duration, types of autoimmune disease, use of glucocorticoid, history of fractures.

Supplementary Table 9. Sensitivity analysis of multiple linear regression of osteoporosis knowledge score and lumbar spine and femoral neck BMD across multiple imputation (MI), complete-case analysis (CCA), and single mean/mode imputation (SI).

| Variables | BMD (g/cm^2^) (MI) | | | |  | BMD (g/cm^2^) (CCA) | | | |  | BMD (g/cm^2^) (SI) | | | |
| --- | --- | --- | --- | --- | --- | --- | --- | --- | --- | --- | --- | --- | --- | --- |
|  | Lumbar spine | | Femur neck | |  | Lumbar spine | | Femur neck | |  | Lumbar spine | | Femur neck | |
|  | *β* (95% CI) | *p*-Value | β (95% CI) | *p*-Value |  | *β* (95% CI) | *p*-Value | β (95% CI) | *p*-Value |  | *β* (95% CI) | *p*-Value | *β* (95% CI) | *p*-Value |
| Osteoporosis knowledge score | 0.003 (0.000, 0.006) | **0.043** | 0.002 (0.000, 0.004) | **0.035** |  | 0.003 (-0.001, 0.006) | 0.101 | 0.003 (0.000, 0.005) | **0.030** |  | 0.003 (-0.001, 0.006) | 0.101 | 0.003 (0.000, 0.005) | **0.030** |
| Osteoporosis knowledge score groups |  |  |  |  |  |  |  |  |  |  |  |  |  |  |
| Quartile 1 | 1[reference] | | 1[reference] | |  | 1[reference] | | 1[reference] | |  | 1[reference] | | 1[reference] | |
| Quartile 2 | 0.026 (-0.013, 0.065) | 0.188 | 0.015 (-0.015, 0.046) | 0.315 |  | 0.017 (-0.027, 0.061) | 0.447 | 0.027 (-0.007, 0.061) | 0.115 |  | 0.017 (-0.027, 0.061) | 0.447 | 0.027 (-0.007, 0.061) | 0.115 |
| Quartile 3 | 0.025 (-0.014, 0.065) | 0.208 | 0.019 (-0.012, 0.050) | 0.221 |  | 0.008 (-0.035, 0.051) | 0.703 | 0.024 (-0.009, 0.057) | 0.131 |  | 0.008 (-0.035, 0.051) | 0.703 | 0.024 (-0.009, 0.057) | 0.131 |
| Quartile 4 | 0.040 (-0.007, 0.086) | 0.096 | 0.034 (-0.002, 0.071) | 0.064 |  | 0.036 (-0.016, 0.087) | 0.174 | 0.045 (0.005, 0.084) | **0.026** |  | 0.036 (-0.016, 0.087) | 0.174 | 0.045 (0.005, 0.084) | **0.026** |
| *P* for trend | 0.118 | | 0.072 | |  | 0.278 | | **0.042** | |  | 0.100 | | 0.057 | |
| Osteoporosis knowledge score groups (LCA) |  |  |  |  |  |  |  |  |  |  |  |  |  |  |
| Low | 1[reference] | | 1[reference] | |  | 1[reference] | | 1[reference] | |  | 1[reference] | | 1[reference] | |
| Moderate | 0.034 (-0.003, 0.071) | 0.074 | 0.028 (-0.001, 0.056) | 0.061 |  | 0.056 (0.016, 0.096) | **0.006** | 0.035 (0.004, 0.066) | **0.027** |  | 0.056 (0.016, 0.096) | **0.006** | 0.035 (0.004, 0.066) | **0.027** |
| High | 0.051 (0.013, 0.088) | **0.008** | 0.027 (-0.002, 0.056) | 0.068 |  | 0.058 (0.015, 0.102) | **0.008** | 0.026 (-0.007, 0.059) | 0.125 |  | 0.058 (0.015, 0.102) | **0.008** | 0.026 (-0.007, 0.059) | 0.125 |
| *P* for trend | **0.010** | | 0.098 | |  | 0.161 | | 0.342 | |  | **0.025** | | 0.093 | |
| Osteoporosis knowledge weight score | 0.010 (0.005, 0.016) | **0.001** | 0.008 (0.003, 0.012) | **0.001** |  | 0.010 (0.003, 0.018) | **0.009** | 0.007 (0.001, 0.013) | **0.018** |  | 0.011 (0.005, 0.017) | **<0.001** | 0.008 (0.003, 0.012) | **0.001** |
| Osteoporosis knowledge weight score groups |  |  |  |  |  |  |  |  |  |  |  |  |  |  |
| Quartile 1 | 1[reference] | | 1[reference] | |  | 1[reference] | | 1[reference] | |  | 1[reference] | | 1[reference] | |
| Quartile 2 | 0.023 (-0.016, 0.061) | 0.250 | 0.009 (-0.021, 0.039) | 0.560 |  | 0.015 (-0.029, 0.058) | 0.506 | 0.002 (-0.032, 0.035) | 0.925 |  | 0.031 (-0.008, 0.069) | 0.122 | 0.012 (-0.019, 0.042) | 0.453 |
| Quartile 3 | 0.041 (0.002, 0.080) | **0.040** | 0.023 (-0.007 0.054) | 0.134 |  | 0.043 (-0.001, 0.088) | 0.055 | 0.020 (-0.015, 0.054) | 0.259 |  | 0.045 (0.006, 0.085) | **0.024** | 0.021 (-0.009, 0.052) | 0.167 |
| Quartile 4 | 0.050 (0.012, 0.089) | **0.011** | 0.033 (0.003, 0.063) | **0.032** |  | 0.044 (0.000, 0.088) | 0.052 | 0.022 (-0.012, 0.057) | 0.197 |  | 0.054 (0.015, 0.092) | **0.006** | 0.031 (0.001, 0.061) | **0.043** |
| *P* for trend | **0.007** | | **0.020** | |  | **0.025** | | 0.117 | |  | **0.005** | | **0.034** | |

Abbreviation: MI, multiple imputation, CCA, complete-case analysis, SI, single mean/mode imputation, LCA, latent class analysis, BMD, bone mass density (gm/cm^2^); β, coefficient; CI, confidence interval.

Osteoporosis knowledge weight score were calculated as the proportion of each logistic regression coefficient (β) relative to the total sum of all βs in the multivariable model predicting low BMD.

Linear regression adjusted for age, sex, BMI, education, occupation, smoking status, alcohol consumption, tea intake, milk consumption, regular calcium intake, sunshine duration, physical activity levels, autoimmune disease duration, types of autoimmune disease, use of glucocorticoid, history of fractures.

Data are presented as β (95% CI) , and *P*-value. *P*-values < 0.05 were marked in bold.

Supplementary Table 10. Sensitivity analysis of multivariable logistic regression of four osteoporosis knowledge score dimensions and the risk of low BMD across multiple imputation (MI), complete-case analysis (CCA), and single mean/mode imputation (SI).

| Variables | Model 3 (MI) | |  | Model 3 (CCA) | |  | Model 3 (SI) | |
| --- | --- | --- | --- | --- | --- | --- | --- | --- |
|  | OR (95% CI) | *P*-value |  | OR (95% CI) | *P*-value |  | OR (95% CI) | *P*-value |
| Osteoporosis knowledge score group regarding symptoms |  |  |  |  |  |  |  |  |
| Tertiles 1 | 1[reference] | |  | 1[reference] | |  | 1[reference] | |
| Tertiles 2 | 0.937 (0.556, 1.583) | 0.806 |  | 0.980 (0.555, 1.737) | 0.944 |  | 0.947 (0.563, 1.597) | 0.836 |
| Tertiles 3 | 0.841 (0.438, 1.607) | 0.600 |  | 0.851 (0.410, 1.752) | 0.662 |  | 0.838 (0.436, 1.603) | 0.594 |
| *P* for trend | 0.640 | |  | 0.676 | |  | 0.636 | |
| Osteoporosis knowledge score group regarding diagnostic method |  |  |  |  |  |  |  |  |
| Tertiles 1 | 1[reference] | |  | 1[reference] | |  | 1[reference] | |
| Tertiles 2 | 1.517 (0.842, 2.761) | 0.168 |  | 1.164 (0.616, 2.211) | 0.640 |  | 1.479 (0.823, 2.684) | 0.193 |
| Tertiles 3 | 2.134 (1.047, 4.409) | **0.038** |  | 1.459 (0.684, 3.139) | 0.330 |  | 2.048 (1.008, 4.213) | **0.049** |
| *P* for trend | **0.028** | |  | 0.262 | |  | **0.035** | |
| Osteoporosis knowledge score group regarding preventive measure |  |  |  |  |  |  |  |  |
| Median 1 | 1[reference] | |  | 1[reference] | |  | 1[reference] | |
| Median 2 | 0.638 (0.397, 1.024) | 0.063 |  | 0.705 (0.414, 1.197) | 0.195 |  | 0.632 (0.393, 1.013) | 0.057 |
| *P* for trend | 0.065 | |  | 0.184 | |  | 0.059 | |
| Osteoporosis knowledge score group regarding risk factors |  |  |  |  |  |  |  |  |
| Tertiles 1 | 1[reference] | |  | 1[reference] | |  | 1[reference] | |
| Tertiles 2 | 0.710 (0.411, 1.221) | 0.216 |  | 0.985 (0.567, 1.716) | 0.957 |  | 0.714 (0.415, 1.226) | 0.222 |
| Tertiles 3 | 0.455 (0.221, 0.927) | **0.031** |  | 0.696 (0.351, 1.368) | 0.294 |  | 0.456 (0.222, 0.926) | **0.031** |
| *P* for trend | **0.032** | |  | 0.318 | |  | **0.031** | |

Abbreviation: MI, multiple imputation, CCA, complete-case analysis, SI, single mean/mode imputation, LCA, latent class analysis, CI, confidence interval, OR, odd ratio.

Osteoporosis knowledge score dimensions: osteoporosis symptoms, diagnostic method, preventive measures and risk factors.

Data are presented as OR, 95% CI, and *P*-value. *P*-values < 0.05 were marked in bold.

Model 3 was adjusted for age, sex, BMI, education, occupation, smoking status, alcohol consumption, tea intake, milk consumption, regular calcium intake, sunshine duration, physical activity levels, autoimmune disease duration, types of autoimmune disease, use of glucocorticoid, history of fractures.

Supplementary Table 11. Sensitivity analysis of multiple linear regression of four OPAAT-C dimensions and lumbar spine and femoral neck BMD across multiple imputation (MI), complete-case analysis (CCA), and single mean/mode imputation (SI).

| Variables | BMD (g/cm^2^) (MI) | | | |  | BMD (g/cm^2^) (CCA) | | | |  | BMD (g/cm^2^) (SI) | | | |
| --- | --- | --- | --- | --- | --- | --- | --- | --- | --- | --- | --- | --- | --- | --- |
|  | Lumbar spine | | Femur neck | |  | Lumbar spine | | Femur neck | |  | Lumbar spine | | Femur neck | |
|  | *β* (95% CI) | *p*-Value | β (95% CI) | *p*-Value |  | *β* (95% CI) | *p*-Value | *β* (95% CI) | *p*-Value |  | *β* (95% CI) | *p*-Value | *β* (95% CI) | *p*-Value |
| Osteoporosis knowledge score group regarding symptoms |  |  |  |  |  |  |  |  |  |  |  |  |  |  |
| Tertiles 1 | 1[reference] | | 1[reference] | |  | 1[reference] | | 1[reference] | |  | 1[reference] | | 1[reference] | |
| Tertiles 2 | 0.006 (-0.029, 0.042) | 0.726 | 0.012 (-0.016, 0.039) | 0.405 |  | -0.002 (-0.041, 0.037) | 0.928 | 0.010 (-0.020, 0.039) | 0.518 |  | 0.006 (-0.030, 0.041) | 0.741 | 0.011 (-0.016, 0.039) | 0.423 |
| Tertiles 3 | -0.007 (-0.051, 0.037) | 0.755 | 0.031 (-0.003, 0.065) | 0.076 |  | -0.009 (-0.058, 0.040) | 0.728 | 0.040 (0.003, 0.078) | **0.036** |  | -0.007 (-0.051, 0.037) | 0.767 | 0.027 (-0.007, 0.061) | 0.124 |
| *P* for trend | 0.756 | | 0.063 | |  | 0.761 | | **0.028** | |  | 0.764 | | 0.115 | |
| Osteoporosis knowledge score group regarding diagnostic method |  |  |  |  |  |  |  |  |  |  |  |  |  |  |
| Tertiles 1 | 1[reference] | | 1[reference] | |  | 1[reference] | | 1[reference] | |  | 1[reference] | | 1[reference] | |
| Tertiles 2 | -0.025 (-0.065, 0.015) | 0.219 | -0.010 (-0.041, 0.021) | 0.525 |  | -0.008(-0.051, 0.036) | 0.730 | 0.006 (-0.027, 0.039) | 0.733 |  | -0.025 (-0.065, 0.016) | 0.229 | -0.010 (-0.041, 0.021) | 0.528 |
| Tertiles 3 | -0.032 (-0.080, 0.016) | 0.196 | -0.019 (-0.056, 0.018) | 0.317 |  | -0.011 (-0.062, 0.041) | 0.688 | -0.005 (-0.044, 0.035) | 0.820 |  | -0.030 (-0.078, 0.018) | 0.218 | -0.015 (-0.053, 0.022) | 0.417 |
| *P* for trend | 0.323 | | 0.306 | |  | 0.751 | | 0.598 | |  | 0.352 | | 0.426 | |
| Osteoporosis knowledge score group regarding preventive measure |  |  |  |  |  |  |  |  |  |  |  |  |  |  |
| Median 1 | 1[reference] | | 1[reference] | |  | 1[reference] | | 1[reference] | |  | 1[reference] | | 1[reference] | |
| Median 2 | 0.024 (-0.008, 0.056) | 0.138 | 0.021 (-0.004, 0.045) | 0.105 |  | 0.007 (-0.028, 0.043) | 0.693 | 0.007 (-0.020, 0.034) | 0.605 |  | 0.025 (-0.007, 0.074) | 0.133 | 0.020 (-0.004, 0.045) | 0.108 |
| *P* for trend | 0.136 | | 0.090 | |  | 0.689 | | 0.533 | |  | 0.130 | | 0.098 | |
| Osteoporosis knowledge score group regarding risk factors |  |  |  |  |  |  |  |  |  |  |  |  |  |  |
| Tertiles 1 | 1[reference] | | 1[reference] | |  | 1[reference] | | 1[reference] | |  | 1[reference] | | 1[reference] | |
| Tertiles 2 | 0.037 (-0.001, 0.074) | 0.055 | 0.015 (-0.015, 0.044) | 0.326 |  | 0.034 (-0.004, 0.073) | 0.077 | 0.003 (-0.026, 0.033) | 0.815 |  | 0.037 (-0.001 0.074) | 0.054 | 0.014 (-0.015, 0.043) | 0.341 |
| Tertiles 3 | 0.046 (-0.001, 0.094) | 0.057 | 0.014 (-0.023, 0.051) | 0.456 |  | 0.039 (-0.006, 0.085) | 0.092 | 0.011 (-0.024, 0.046) | 0.544 |  | 0.047 (-0.001, 0.094) | 0.054 | 0.016 (-0.020, 0.053) | 0.385 |
| *P* for trend | 0.062 | | 0.459 | |  | 0.074 | | 0.520 | |  | 0.058 | | 0.393 | |

Abbreviation: OPAAT-C: Chinese Version of Osteoporosis Prevention and Awareness Tool, MI, multiple imputation, CCA, complete-case analysis, SI, single mean/mode imputation, LCA, latent class analysis, BMD, bone mass density (gm/cm^2^); β, coefficient; CI, confidence interval.

OPAAT-C dimensions: osteoporosis symptoms, diagnostic method, preventive measures and risk factors.

Linear regression adjusted for age, sex, BMI, education, occupation, smoking status, alcohol consumption, tea intake, milk consumption, regular calcium intake, sunshine duration, physical activity levels, autoimmune disease duration, types of autoimmune disease, use of glucocorticoid, history of fractures.

Data are presented as β (95% CI) , and *P*-value. *P*-values < 0.05 were marked in bold.

Supplementary Table 12. Construction of the osteoporosis action score.

| Items | Coding criteria | Logistic regression *β* | Standardized weight |
| --- | --- | --- | --- |
| Smoking status | Yes = 0, No = 1 | -0.525 | 7.500 |
| Alcohol consumption | Yes = 0, No = 1 | 0.303 | -4.471 |
| Tea intake | <1 cup/week = 1, ≥1 cup/week = 0 | 0.129 | -1.843 |
| Milk consumption | ≥1 cup/week = 1, <1 cup/week = 0 | -0.288 | 4.114 |
| Regular calcium supplementation | Yes = 1, No = 0 | 0.337 | -4.814 |
| Sunshine duration | Others = 1, Almost no sunlight = 0 | -0.128 | 1.829 |
| Physical activity levels | ≥3,000 MET-min/week and ≥7 d/w of PA at three intensities = 1, else = 0 | 0.092 | -1.314 |

Standardized weights were calculated as the proportion of each logistic regression coefficient (β) relative to the total sum of all βs in the multivariable model predicting the risk of low BMD.

Smoking status was categorized as current ("yes") and former/never ("no"). Alcohol consumption was categorized as current ("yes") and former/never ("no"). Tea intake and milk consumption were defined as ≥1 cup/week and <1 cup/week. Regular calcium intake was defined as either calcium supplementation or dietary consumption occurring ≥3 times per week. Sunshine duration was divided into two categories: almost no sunlight (very little sun exposure) and others (sunlight exposure 30-60 minutes per day and >60 minutes per day). Physical activity levels, assessed using the IPAQ-SF, were classified as low (<3000 MET-min/week and <7 d/w of PA at three intensities) and high (≥3,000 and ≥7), based on self-reported duration and intensity.

Logistic regression was adjusted for age groups, sex, and BMI groups, autoimmune disease duration, use of glucocorticoid and history of fractures.
